# The Minimum Inhibitory Concentration of Antibiotics: Methods, Interpretation, Clinical Relevance

**DOI:** 10.3390/pathogens10020165

**Published:** 2021-02-04

**Authors:** Beata Kowalska-Krochmal, Ruth Dudek-Wicher

**Affiliations:** Department of Pharmaceutical Microbiology and Parasitology, Faculty of Pharmacy, Medical University of Silesian Piasts in Wroclaw, Borowska 211, 50-556 Wroclaw, Poland; r.dudek.wicher@gmail.com

**Keywords:** minimal inhibitory concentration, antibiotics, dilution and gradient methods, interpretation of results, the importance of MIC value

## Abstract

Inefficiency of medical therapies used in order to cure patients with bacterial infections requires not only to actively look for new therapeutic strategies but also to carefully select antibiotics based on variety of parameters, including microbiological. Minimal inhibitory concentration (MIC) defines in vitro levels of susceptibility or resistance of specific bacterial strains to applied antibiotic. Reliable assessment of MIC has a significant impact on the choice of a therapeutic strategy, which affects efficiency of an infection therapy. In order to obtain credible MIC, many elements must be considered, such as proper method choice, adherence to labeling rules, and competent interpretation of the results. In this paper, two methods have been discussed: dilution and gradient used for MIC estimation. Factors which affect MIC results along with the interpretation guidelines have been described. Furthermore, opportunities to utilize MIC in clinical practice, with pharmacokinetic /pharmacodynamic parameters taken into consideration, have been investigated. Due to problems related to PK determination in individual patients, statistical estimation of the possibility of achievement of the PK/PD index, based on the Monte Carlo, was discussed. In order to provide comprehensive insights, the possible limitations of MIC, which scientists are aware of, have been outlined.

## 1. Introduction

The increasing resistance of bacteria to antibiotics and more and more common failed infection treatments call for identification of the underlying causes of this problem and, further, for searching the ways to reduce it and to improve the effectiveness of infection therapies. One of the recognized reasons behind failed therapies is the drug selection pressure, especially when they are ill-chosen and administered in doses too small, which leads to the survival of a resistant bacterial population or induces antibiotic resistance mechanisms [1]. Therefore, it is very important to use antibiotics in true bacterial infections and in such doses so as to increase the likelihood of therapeutic effectiveness. During initial phases of infections, especially severe ones, empirical therapy is usually undertaken, where an antibiotic is selected depending on the location of the infection, the patient’s clinical condition, therapeutic history, concomitant diseases and organ dysfunctions. The antibiotic should be effective against probable pathogens, whose frequency of isolation and drug sensitivity should be known from epidemiological data obtained from a retrospective analysis of the results of multiple microbiological studies. Whenever possible, the administration of an antibiotic in empirical therapy should be preceded by sampling for microbiological tests, and the results of such tests should be the basis for verification of the validity of preliminary therapeutic decisions and for the application of a targeted therapy. Thus, in both empirical and targeted therapy, microbiological test results serve as a strong support for the choice of the optimal antibiotic. The identification of the pathogen, sometimes also including the determination of its quantity per 1 g or mL of the sample, combined with the results of analytical and clinical studies, is the basis for the definitive diagnosis of the infection. An antibiogram, on the other hand, suggests the choice of a drug expected to be clinically effective in bacterial infections. Commonly, antibiograms contain a qualitative assessment of a strain’s susceptibility or resistance to antibiotics as well as information about the detected resistance mechanisms. For many infections, such parameters are sufficient to stop the use of antibiotics already administered in case of the strain’s resistance and to replace it with a drug to which the strain is susceptible. However, in the case of seriously ill patients, who suffer from chronic infections, who have been treated with a broad range of antibiotics, and who have a history of failed therapies, much more precise guidance is needed to facilitate the selection of an optimal (i.e., effective) antibiotic. Among such microbiological parameters is the minimum inhibitory concentration (MIC) of the antimicrobial. This value has been known for a very long time. For many years it has been determined only occasionally but now it has been appearing increasingly frequently in the results of routine tests. However, the ability to use it for effective and optimal therapy is still limited and sometimes, despite much higher costs incurred than in qualitative methods, it is completely unused.

## 2. Definition and Methods

### 2.1. What is MIC?

MIC is the lowest concentration of an antibacterial agent expressed in mg/L (μg/mL) which, under strictly controlled in vitro conditions, completely prevents visible growth of the test strain of an organism [2].

### 2.2. MIC Determination Methods

The following methods are used:

1. Dilution methods
  - in agar
  - in a liquid medium
    ○ micromethod/ microdilution
    ○ macromethod/ macrodilution
2. Gradient methods
  - strips impregnated with a predefined concentration gradient of antibiotic

#### 2.2.1. Dilution Methods

EUCAST [3] mostly recommends broth microdilution, with the exception of fosfomycin and mecillinam for which it recommends agar dilution. The American CLSI [4], on the other hand, admits interchangeable use of broth and agar dilution for most bacteria and antibiotics. The exceptions are *H. influenzae* strains and antibiotics colistin and daptomycin for which MIC can be determined only by broth dilution and fosfomycin for which, like in EUCAST guidance documents, MIC can be measured using the agar dilution method. In addition, CLSI recommends HTM medium for *H. influenzae* instead of the MH–F broth medium recommended by EUCAST.

To determine MIC values, all quantitative methods use Mueller–Hinton (MH) medium either in the form of agar (MHA) or broth (MHB), in some cases additionally supplemented with, for example, 5% lysed horse blood or other compounds depending on bacteria or antibiotic type (Table 1). Only for anaerobic bacteria Brucella agar with Hemin (5 µg/mL), Vitamin K (1 µg/mL) and 5% lysed horse blood is used [4,5].

To determine MIC by dilution methods, antibiotics are also needed in a substance that require preliminary dissolution to obtain a stock solution and then dilution to obtain an appropriate starting concentration. For most antibiotics, water is both a solvent and a diluter, including for most beta-lactams, fluoroquinolones and aminoglycosides. Some require alcohol as a solvent, especially macrolides, chloramphenicol and rifampicin, while others require a phosphate buffer or dimethyl sulfoxide DMSO (Table 2). Dissolved and diluted antibiotics are used to make working solutions in Mueller-Hinton broth or agar [6,7].

Working solutions should contain double dilutions of antibiotics, with the range of concentrations used for testing depending on the medication concerned and should take into account the MIC breakpoints for reference strains [6]. Subsequent double dilutions of the antibiotic should be performed using the schemes available in the documents [6] and proposed by EUCAST [8].

In the broth microdilution method, the prepared working solutions with double dilutions of antibiotics are distributed into appropriate wells of microtiter plates, and in this form they can be either used directly for MIC determinations or can be stored in plastic bags for up to three months at a temperature ≤ −60 °C [6]. Tigecycline is an exception for which MIC testing should take place within 12 h of the preparation of the MHB medium. This is due to the fact that over time, the medium accumulates oxygen, which in turn reduces the activity of tigecycline [3,4,6,9].

In the agar dilution method, each of the obtained antibiotic concentrations at a volume of 1 mL is added to 19 mL of still liquid MHA medium at a temperature of 45–50 °C and is poured on Petri dishes with a diameter of 9 cm [8].

##### Bacterial Inoculum

The bacterial suspension should be prepared from morphologically similar colonies cultured overnight on a nonselective solid or liquid medium. The inoculum to be treated with subsequent dilutions of the antibiotic should have the following final values in the respective methods:

- broth microdilution method 5 × 10^5^ CFU (colony forming units) /mL [6]
- agar dilution method 1 × 10^4^ CFU/spot [7,8]

To obtain any of the above bacterial suspensions, a suspension of 0.5 McFarland should be prepared first.

To obtain a bacterial suspension with a density of 5 × 10^5^ CFU/mL for the purposes of broth microdilution, 0.5 McFarland suspension should be diluted 100× to a density of 10^6^ CFU/mL (9.9 mL broth + 0.1 mL 0.5 McFarland suspension) and then poured to wells containing the appropriate antibiotic concentrations in the broth (50 μL bacterial inoculum + 50 liquid medium with antibiotic or 10 μL inoculum to 100 μL diluted antibiotic). If commercial tests with a freeze-dried antibiotic are used in the wells, a suspension of 5 × 10^5^ should be obtained immediately by adding 50 μL of 0.5 McFarland suspension to 10 mL of the broth). *S. pneumoniae* requires a transfer of 100 μL of 0.5 McFarland suspension to 10 mL broth to obtain a final inoculum 5 × 10^5^ CFU/mL [10].

For the agar dilution method, the final inoculum 1 × 10^4^ CFU/spot is obtained by diluting the 0.5 McFarland suspension 10× in NACL or broth and spotting 1 μL of such suspension on the MHA media with subsequent antibiotic dilutions [8].

Within 30 min of preparation, the inoculum should be added to the liquid media or placed on solid media with the antibiotic so that cell density (CFU/mL) is maintained. The tests should be incubated at 35 ± 1 °C for 18–24 h (full 24 h is required especially for glycopeptides and for oxacillin [6] as well as for testing *Streptococcus* spp. and *Haemophilus* spp. strains [10]. Incubation should be carried out under aerobic conditions. Only in exceptional cases, for strains such as *Neisseria* spp., incubation is conducted in an atmosphere enriched with 5% CO_2_ [8] or for anaerobic bacteria it is done under anaerobic conditions and for 48 h [5].

Bacterial inoculum should be controlled as it has a major impact on the reliability of MIC tests. The obtaining of 0.5 McFarland suspension is controlled by measurements in a densitometer or spectrophotometer, where absorbance at a wavelength of 625 nm should be in the range from 0.08 to 0.13 [9,11]. The inoculum obtained in microtiter plate wells should also be controlled. For this purpose, when using broth microdilution, 10 μL should be sampled from the growth control well (MHB medium with bacterial suspension and without antibiotic) and added to 10 mL of broth or salt, and then 100 μL from such dilution should be placed on a solid medium, well spread on the MHA medium and incubated for 12–18 h at 35 ± 1 °C. Obtaining of a growth of 20–80 colonies of a given bacterial strain proves the density of 5 × 10^5^ CFU/mL [6].

In each of the methods, quality control needs to be carried out by controlling medium sterility, strain growth and quality of the results obtained by assessing the MIC of the tested antibiotic for reference strains whose list is given in Table 1. The reference strains listed in Table 1 are recommended by both EUCAST and CLSI [3,4]. The obtained MIC values for reference strains should be within the range of concentrations recommended by EUCAST and CLSI [4,12].

##### Reading of Results

The MIC value is the lowest concentration of an antibiotic at which bacterial growth is completely inhibited. In the agar dilution method, growth of 1–2 colonies or faint haze are disregarded [7,8]. In the broth microdilution method, for certain antibiotics, separate rules for reading the MIC value are used [13], including for the following:

- bacteriostatic antibiotics against Gram-positive bacteria (chloramphenicol, tetracycline, clindamycin, erythromycin, linezolid, tedizolid) and against Gram-negative organisms (tygecycline, eravacycline): disregard pinpoint growth at the bottom of the well
- trimethoprim-sulfamethoxazole for all bacteria: read the MIC at the lowest concentration that inhibits ≥80% of growth as compared to the growth control.

To facilitate reading in the broth microdilution method, resazurin (a weakly fluorescent blue dye) can be used, which is reduced by active bacteria to fluorescent resorufin (pink) [14]. Tests in which no growth is observed at lower antibiotic concentrations and visible growth of bacteria is observed at higher concentrations need to be repeated. This may be due to several reasons, including technical errors associated with, e.g., inappropriate antibiotic dilution. However, the reason for Eagle’s effect is much more engaging. It refers to bacteria, which paradoxically have increased ability to survive in the presence of higher than optimal bactericidal concentration of antibiotic [15]. This phenomenon was first described by scientist Harry Eagle in 1948 with regard to penicillin. Currently, Eagle effect is found for a number of antibiotics like beta-lactams, glycopeptides, fluoroquinolones, aminoglycosides, polymyxins, rifampicin. It is also observed in case of various micro-organisms, including *Staphylococcus* spp., *Streptococcus* spp., *Enterococcus* spp., and Gram-negative, in particular beta-lactamases-positive bacteria. Interestingly, it does not occur neither for beta-lactamases-negative bacteria nor in a presence of beta-lactam inhibitors. In clinical settings, Eagle effect leads to treatment failure due to application of excessive dose of antibiotic. Studies have shown that vancomycin concentrations of 20 × MIC or higher applied against *E. faecalis* or *Clostridium difficile* have contributed to bacteriostatic rather that bactericidal effect of glycopeptide. In contrast, concentrations up to 9 × MIC were effective and resulted in positive clinical outcome [16,17]. The mechanisms underlying the Eagle effect are not fully understood. The Scientific attention is focused on possible increased production of beta-lactamases due to high concentration of antibiotic, diminished expression of PBPs (penicillin binding proteins) in the stationary phase of bacteria or oxidative stress induced by quinolones [15].

#### 2.2.2. Gradient Method

MIC Determination Using the Gradient Method is much less complicated than dilution methods. The use of E-test strips impregnated with a predefined gradient of antibiotic concentrations makes the method simple, fast and applicable in routine microbiological diagnostics. Unfortunately, in recent years, the use of the disc-diffusion method using strips has been significantly reduced. It turned out that it produces unreliable determinations of sensitivity to colistin and vancomycin in Staphylococcus spp. strains or to fosfomycin [3,4]. In the case of colistin, this method usually produces lower MIC values than the reference broth microdilution method. Hence, it does not usually allow for identification of resistant strains [18,19,20]. The above is explained both by the size of the polymyxin molecule (which is the reason why it has limited diffusion possibilities from the strip) and by a possible interaction with the plastic of which the strip is made [21,22]. In May 2019, a warning was also issued on the EUCAST website regarding the possibility of obtaining false negative results when using gradient tests in the evaluation of vancomycin resistance of strains of the genus *Enterococcus* spp. and in the assessment of sensitivity of *Streptococcus pneumoniae* to benzylpenicillin [23,24]. The rejection of this method for the determination of MIC of certain antibiotics has ultimately led to a reduction in the issuance of results including an assessment of susceptibility to colistin or to the increasingly common fosfomycin. It has not been replaced by dilution methods because most laboratories are unable to implement them in their routine work. Therefore, research is ongoing to assess the suitability of strips in bacterial drug sensitivity testing taking into account the impact of various factors on the results. In recent years, there have been reports that enhancing the MHA media with calcium could improve the reliability of colistin MIC determination. In their studies, Gwoździński et al. [25] showed a high percentage of essential agreement (EA) of the MIC value determined using the gradient method on calcium-enhanced medium as compared to the reference method (broth microdilution) but only for colistin-sensitive strains. EA for resistant strains was much lower (13%), although categorical agreement (CA) was higher and stood at 87%. A recent research study from the United States published in 2020 [26] indicates that calcium supplementation does not bring the expected improvement in the reliability of the gradient method in the determination of colistin MIC, as EA and CA were 65.5% and 73.7% respectively for all Gram-negative organisms tested, including those susceptible and resistant to colistin. Similarly, work is ongoing to assess whether the gradient test actually does not allow a reliable assessment of fosfomycin MIC. Studies carried out so far offer differing results. According to some authors, MIC values determined using test strips are higher than those obtained using the reference method, so Fosfomycin-resistant strains [27,28] are found more often when strips are used as a test method. Flam et al. suggest that strains found to be resistant using test strips should be verified by the agar dilution test [25]. In another study, Italian authors found 100% CA between the gradient and reference methods for *E. coli* ESBL(+) and *Klebsiella pneumoniae* NDM/OXA-48 [29]. However, currently, the gradient test is not allowed for fosfomycin MIC determinations by American regulators [4] and EUCAST does not recommend it as a reference method either [3], although some national recommendations (like, e.g., the Polish ones) permit its application [30].

The gradient method requires a 0.5 McFarland suspension for all types of bacteria except the suspension obtained from *Streptococcus pneumoniae* grown on a chocolate agar plate for which 1 McFarland suspension should be used [3]. Reading the MIC value using the E-test gradient strip is more complicated than performing the test itself. It may depend on the tested bacterial strain, the antibiotic (in particular whether it is bacteriostatic or bactericidal), the resistance mechanism, the presence of a heterogeneously resistant population and even on the way in which the E-test gradient strip test is performed. Therefore, MIC assessments should be carried out in accordance with the manufacturer’s instructions which take account of such factors [31]. The above-mentioned fosfomycin can be an example here, for which the growth of individual colonies in the growth inhibition zone should not be taken into account in determining MIC value [32].

## 3. Interpretation of MIC

The determined MIC value must be compared with MIC clinical breakpoints to assess whether the strain is susceptible or resistant to the antibiotic. Evaluation of antibiotic resistance based on the MIC value does not mean the identification of the resistance mechanism. However, due to epidemiological reasons, qualifying such a strain to resistant category according to the MIC value may be a trigger to undertake further research on the detection of resistance mechanism. For this purpose, molecular or phenotypic methods are used, including tests such as Carba-NP for the detection of carbapenemases or Double Disc Synergy Test for ESBL enzymes in Gram-negative bacteria. Clinical breakpoints are currently set and published primarily by two organizations in the world: the European EUCAST (European Committee on Antimicrobial Susceptibility Testing) and the American CLSI (Clinical and Laboratory Standards Institute), and partly by the FDA (Food and Drug Administration) [3,4]. The determination of clinical breakpoints (BP) requires the cooperation of specialists in various fields: microbiologists, pharmacologists, infectious diseases physicians, but also experts in data processing and statistical analysis [33,34]. BP determination requires taking into account antibiotic doses including the maximum doses for which these values will be established, clinical indications for which they will be applied and reference to the specific micro-organisms. In the course of determining BP, the distribution of MIC values for wild strains, i.e., those which do not have any resistance mechanism to the test antibiotic, is assessed and an epidemiological cut-off value (ECOFF) is determined, i.e., the highest MIC typical for wild-type strains. ECOFF distinguishes between bacterial strains without any phenotypically established acquired antibiotic resistance mechanism and those displaying such mechanisms [35,36,37]. The presence of resistance mechanisms to various antibiotics is also subject to phenotypic and genotypic evaluation. Among very important steps is the analysis of pharmacokinetic/dynamic parameters (the fate of drugs in the body and their activity against bacteria) in preclinical and clinical trials [34,38]. Literature reports also need to be analyzed and clinical breakpoints for MIC need to be finally set but in such a manner so that they do not separate MIC values for wild type strains [34]. MIC clinical breakpoints in turn are used to establish the clinical breakpoints for the disc-diffusion method correlating with the BP for MIC. The clinical MIC breakpoints for susceptible strains are usually not equal to the epidemiological cut-off values for susceptible strains. The former is most often higher and includes not only wild-type strains but also those with low levels of resistance, which, however, does not affect clinical efficacy [1]. In addition, the established values are not constant, so they are updated periodically, as microbes and dosing rules change. It should also be remembered that the interpretation of drug sensitivity tests depends on the recommendation adopted in a given country or region (EUCAST, CLSI, FDA) and unfortunately sometimes it varies despite the fact that the same MIC value has been obtained in the study. Therefore, when comparing the incidence of susceptible and resistant strains across the world, it is necessary to take into account the impact of recommendations on cumulative data.

## 4. The Importance of MIC Values in Clinical Practice

Like the bacterial growth inhibition zone in the qualitative method, the MIC value serves as the basis for assessing the category of susceptibility or resistance of the pathogen to a given antibiotic. According EUCAST [39] recommendations, two susceptibility categories and one resistance category have been introduced since 2019-01-01:

- Susceptible (S), standard dosing regimen: there is a high likelihood of therapeutic success using a standard dosing regimen of the agent.
- Susceptible (I), increased exposure: there is a high likelihood of therapeutic success because exposure to the agent is increased by adjusting the dosing regimen or by its concentration at the site of infection.
- Resistant: there is a high likelihood of therapeutic failure even when there is increased exposure.

The major shift in the clinical interpretation of results, concerns those bacterial strains classified by the end of 2018 as intermediate susceptible to antibiotics (I). Such strains were previously included in the epidemiological reports as resistant. From a clinical point of view the modification of the “I” definition implicates a significant change in results interpretation.

Both, an antibiotic with the new susceptibility category –I and one with the -S category, contributes to the same degree of clinical efficacy.

In order to achieve therapeutic success with category “I”, high doses of the antibiotic should be used, the dosing interval should be reduced or the administration route should be changed, (continuous or prolonged infusions should be applied for example). The option selected to increase exposure depends on the type of antibiotic and its pharmacokinetic/pharmacodynamic parameter (PK/PD) (discussed below in this review). However, EUCAST has prepared a table with the dosing rules taking into account standard dosing for category S and high doses for the new category I (doses were updated in 2021,version 11.0). This facilitates the decision-making with regard to dosing or with regard of the method of drug administration. Based on new rules of MIC values interpretation, the use of, e.g., high doses of ciprofloxacin, i.e., 0.75 g × 2 oral or 0.4 g × 3 i.v., may contribute to the eradication of *Pseudomonas aeruginosa* strain with susceptibility category I to this antibiotic (0.001 < MIC < 0.5 mg/L) [40]. Wantia et al. have shown that the knowledge of new criteria for clinical interpretation, especially concerning category I strains, among healthcare professionals has to be improved immediately [41]. Proper clinical interpretation will result in inclusion of antibiotics with category (I) to therapeutic possibilities, therefore, there is an increased need of healthcare professionals training on newly introduced rules of MIC values clinical interpretation.

For some antibiotics and bacteria, the determination of MIC is the only reliable phenotypic method for assessing drug sensitivity because qualitative methods have provided false results (Table 3) [3,4]. Reason might be poor penetration of an antibiotic into agar, like it happens in case of polymyxin or daptomycin; alternatively, due to impossible distinctions between wild type isolates and those with non-vanA-mediated glycopeptide resistance in *Staphylococcus* spp. For anaerobic bacteria false results may occur due to nondefined breakpoints [40,42,43]. With regard to daptomycin, it is also difficult to obtain appropriate concentration of calcium ions in agar, which are necessary for the evaluation of bacterial susceptibility. All of the facts mentioned above make the disc-diffusion method not recommended [44].

In addition, contrary to qualitative methods, the MIC value allows to assess the degree of susceptibility or resistance to the antibiotic. Information on the degree of susceptibility carries great epidemiological and clinical value, but it must be properly interpreted. The differences in the degree of a strain’s susceptibility to antibiotics cannot be assessed by making a direct comparison of the MIC values obtained for such antibiotics, which, unfortunately, is sometimes done. Such an interpretation leads to the erroneous belief that the strain is most sensitive to the antibiotic for which the MIC is the lowest. EUCAST has greatly facilitated the assessment of susceptibility degree of micro-organisms to antibiotics by introducing new criteria for result interpretation. These criteria distinguish between two levels of susceptibility of strains. The first level concerns standard dosing whereas the second level is referred to higher MIC values and requires increased exposure to the antibiotic. Of course, the magnitude of the MIC value will have an impact on the probability of the pharmacokinetic/pharmacodynamic indexes which are crucial for therapy efficacy. It must not be forgotten that in some cases higher levels of antibiotic MIC close to breakpoints may indicate therapy failure, although the strain might be considered as sensitive at standard doses. This may be the first signal of resistance to the medicine. Such a phenomenon was found, for example, in *Salmonella enterica* serovar Typhi (*S. Typhi*). Strains with MIC ≤ 0.06 mg/L had a point mutation associated with the *gyrA* gene, which further led to the development of resistance of this strain to fluoroquinolones [42,45]. The relationship between the MIC value of susceptible strain and the effectiveness of the medicine is also described when vancomycin is used in *S. aureus* infections. According to literature data [46] and EUCAST guidelines [40], a MIC of 2 mg/L for vancomycin carries the risk of therapy failure despite the strain is classified as susceptible in compliance with accepted breakpoint.

Why does the degree of susceptibility matter? The more susceptible the strain to the antibiotic, the greater the likelihood that its MIC is below the ECOFF and therefore the strain does not develop any drug-resistant subpopulation, so there is no increased risk of bacterial survival during treatment. In addition, high susceptibility of the strain increases the chance for reaching the therapeutic concentration of the antibiotic and for effective eradication of the pathogen using the standard dosage even in patients with significant changes in pharmacokinetic parameters. The knowledge of the degree of susceptibility of strains to various antibiotics used in the hospital may also be a tool used in antibiotic stewardship [47,48,49,50]. Thus, antibiotics whose MIC value for most strains (MIC_90_) is close to the breakpoint could be transferred to a group of medications with a limited access to empirical therapy, unless of course there are other factors in favor of using such an antibiotic. Doing so could contribute to reducing the selection of antibiotic-resistant strains, although this would need to be confirmed in the results of studies which are difficult to perform. It is virtually impossible to obtain data about the distribution of MIC values of antibiotics in most hospitals, because the actual values of MIC (and not the approximate MIC values offered by automated systems such as VITEK or Phoenix) are not routinely determined and if they are, they are performed for selected antibiotics only. Thus, there is usually no cumulative data on the distribution of MIC values for dominant pathogens in specific infections. However, this does not mean that the antibiotic stewardship team cannot introduce, in consultation with the microbiological laboratory, a program to monitor the degree of susceptibility to certain antibiotics in a given hospital, especially in those units where, despite the strain’s antibiotic susceptibility, therapeutic failures are more common. The obtained data could be used as a basis for assessing whether the list of antibiotics dedicated for empirical therapy and the dosage regimens adopted are appropriate for the degree of susceptibility of the strains. Based on such cumulative data, Kuti et al. introduced prolonged infusions of meropenem and continuous infusions of cefepime in an intensive care unit to achieve effectiveness given the high MIC values of these antibiotics [47]. In addition, by establishing MIC values in the range 1.5–2 mg/L for most MRSA strains, they enabled the introduction of linezolid to therapy if a three-day high-dose vancomycin therapy did not improve the patient’s clinical outcome. After 12 months, this procedure contributed to reducing mortality from 21.6% to 8.5% and hospitalization time from 23 days to 10.5 days [47]. The introduction of such solutions simply by transferring them from other hospitals without any reference to the local epidemiological situation is unjustified and will not produce the expected results.

The use of low MIC values antibiotics during infections treatment may improve therapy efficacy. However, it should be taken into consideration that in some cases, even when correct dosing is applied, pathogen eradication will not be achieved. There may be various reasons of such a condition including heterogeneous antibiotic resistance (heteroresistance), tolerance or persistence of micro-organisms in the environment of certain antibiotics. Each of these phenomena is different from commonly described antibiotic resistance. Typical bacterial resistance is displayed in many mechanisms originated from stable genetic mutation or as a result of expression of acquired, by entire cell population, resistance genes. Heteroresistance means the resistance of a very small subpopulation of cells, which begins to grow rapidly in the presence of an antibiotic while the vulnerable population is killed. Antibiotic discontinuation reduces replication of a resistant subpopulation [51]. The detection of resistant cells in the tests is usually not possible due to the low frequency of their occurrence in the population, which may lead to wrong conclusions about antibiotic susceptibility. Sometimes, such a heterogeneous subpopulation may be observed in gradient tests as a presence of bacterial colonies in a growth-inhibition zone. The detection of such strains usually requires an analysis of the population profile—which is not performed routinely. Heteroresistance was described for bacteria, such as *S. aureus, Klebsiella claceae* (formerly *Enterobacter cloacee*), *Klebsiella pneumoniae*, *E. coli*, *P. aeruginosa*, *Accinetobacter* spp., and in many antibiotics, especially colistin, fosfomycin or even carbapenemes. Difficult detection of heteroresistance to colistin, one of the last active antibiotic against resistant Gram-negative bacteria, has led to recommendations update. It is advised to administer properly It is advised to administer properly adjusted first loading dose of colistin and frequently use it in combination with other antibiotics [52]. Moreover, Fernandez et. al. reported between 20–24% of strains with subpopulation heteroresistant to imipenem and meropenem [53]. This is not only a diagnostic problem but above all a clinical plight which causes up to 10% failure in treatment despite in vitro strains susceptibility [51]. Another phenomenon is tolerance, which means the survival of entire population of a strain in a presence of high antibiotic concentration despite the absence of cells resistant to antibiotic. Bacteria with such a tolerance do not grow or divide but stay viable. The tolerance may arise from genotype and be associated with mutation that enables bacteria to avoid bactericidal activity of antibiotic. It may also be of phenotypic character when growth inhibition or retardation is a result of poor nutritional conditions. The tolerance applies only to bactericidal antibiotics. Its bactericidal activity decreases or disappears while bacteriostatic properties are maintained. Bacteria tolerating and these nontolerating antibiotics can have the same MIC. The strain is considered to display tolerance when an antibiotic at a concentration 32 times higher than the MIC does not contribute to 99.9% reduction in the number of bacteria used in the test (Minimal Bactericidal Concentration MBL / MIC > 32 mg/L) [51,54]. Persistence is yet another bacterial mechanism that can contribute to ineffective therapy despite the apparent strain sensitivity. Survival concerns only a small bacterial subpopulation (1%) and is based on the presence of temporarily inactive or very slow-dividing cells [51,54]. Those three phenomena discussed above are very difficult to detect and may be the explanation of treatment inefficacy. Subsequently, survival of the bacterial population despite antibiotic presence can promote the spread of resistance mechanisms.

The MIC value is regarded to have the greatest importance in the optimization of targeted antibiotic therapy. For this purpose however, it must be analyzed together with pharmacokinetic (PK) parameters that describe the fate of the drug in the host organism. The most important PK parameters are: volume of distribution (Vd), elimination half-life (t_1/2_), clearance (CL), maximal concentration (Cmax), minimal concentration (Cmin), and area under curve (AUC). The value of these parameters depends on many factors, such as the patient’s weight or age and on the degree of organ dysfunction, the supply of fluids, but it also varies in different groups of patients. In addition, PK parameters change with time. In the case of antibiotic-resistant strains, identification of the mechanism of bacterial resistance, may have an additional impact on the significance of the MIC value. This topic is particularly discussed in case of Gram-negative bacteria producing carbapenemases. It is widely known that carbapenemases may possess a different spectrum and strength of hydrolytic activity against particular beta-lactams, including carbapenems. According to both EUCAST and CLSI, the detection of the resistance mechanism does not eliminate the possibility of carbapenems use in the treatment of infections caused by carbapenemase-producing bacteria. Regardless of the type of carbapenemase or extended spectrum beta-lactamases (ESBL) produced, clinical interpretation and S, I or R classification of the strain should be based on the obtained MIC value. Currently, meropenem is considered the most important carbapenem, active against Gram-negative *Enterobacterales*. It is listed among antibiotics recommended for the treatment of infections caused by CPE strains, especially when the strain is defined as S (MIC ≤ 2 mg/L, EUCAST BP)). Usually, elicitation of full susceptibility indicates effective therapy with the use of standard doses of a drug, but in this case, due to detection of carbapenemase production, meropenem should be used in high doses, i.e., 2 g every 8 h in prolonged infusions. In a range of values 2 mg/L < MIC ≤ 8 mg/L (Susceptible (I), increased exposure, according to EUCAST v.11.0), meropenem should be used in not only in high doses and with prolonged infusion but additionally in combination with such antibiotics as colistin, tigecycline or amikacin, depending on susceptibility of the pathogen. If the MIC value does not exceed 32 mg/L, it is also allowed to use meropenem in case of resistance to carbapenem [55]. With 8 < MIC values ≤ 32 mg/L, it is necessary not only to apply high doses, but also to combine it with at least two other antibiotics [56]. It has been shown that combination therapy with meropenem, contributed to an increase in patients’ survival, especially with MIC ≤ 8 mg/L (16/19 patients with critical infections). It should be noted that toward KPC(+) strains, new antibiotics with activity against specific carbapenemase should be used, such as ceftazidime with avibactam or meropenem with varbobactam. The most difficult selection of antibiotics is when pan drug resistance is identified. This phenomenon is mostly observed in case of NDM(+) strains that acquire other resistance mechanisms. In that case, the only solution is a combination therapy with minimum of three drugs. Does the MIC of the antibiotics matter in such a case? The authors of the International Consensus Guidelines for the Optimal Use of the Polymyxins from 2019 [52] suggest to choose drugs with the lowest degree of resistance, where MIC value is closest to the breakpoints. Therefore, even in extreme situations, the MIC value may be important, although there is no evidence of the effectiveness of such a procedure.

The quantitative relationship between MIC and PK is determined by the pharmacokinetic/pharmacodynamic indices PK/PD [57,58,59,60,61,62,63,64]. The clinical efficacy of different groups of antibiotics depends on one of three different PK/PDs:

(1) T > MIC, a parameter reflecting the percentage (%) of time between consecutive administrations of an antibiotic in which the drug’s concentration remains above the MIC. The closer the T > MIC parameter is to 100% the greater the likelihood of efficacy. The achievement of this value is especially important in immunosuppressed patients and in the case of Gram-negative infections [63,65,66]. For carbapenems (due to the prolonged effect) and for Gram-positive organisms, the required T > MIC threshold may be lower, as shown in Table 4 [63,67,68,69]. It is easy to deduce that the lower the MIC value of an antibiotic, the easier it is to achieve the required parameter with a standard dosage. In the case of higher MIC values, it may be necessary to reduce the dosing range, e.g., from every 8 h to every 6 h or every 4 h or to use continuous or prolonged infusions depending on the summary of product characteristics [69,70], or to choose another antibiotic for which the PK/PD index will be achieved. Many authors believe that this parameter should be presented in a more detailed form, i.e., ∫T > 4 × MIC, due to the fact that the concentration of the antibiotic should be 4–5 times the MIC to be therapeutic and in addition, only the concentration of the free fraction of the antibiotic is relevant (unbound to plasma proteins (∫)) for the therapy to be effective [60,71]. For antibiotics whose action is dependent on T > MIC, maximization of the dose above 4–5 × MIC is not relevant for the efficacy of the therapy [66,72].

**Figure 1:**
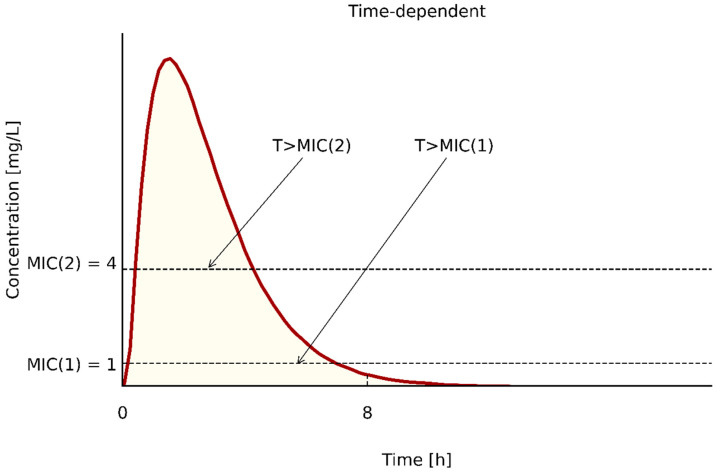
The time above the MIC for a dosing period.

It becomes easier to capture the relationship between the MIC and the achievement of the PK/PD index when the interaction is presented in a graphic form (Figure 1) and also with one of the mathematical formula for the calculation of T > MIC proposed by Turnidge in 1998 [68]:

(1) $${\%T}_{C>\mathrm{MIC}}\;=\ln\left[\frac{\mathrm{Dose}}{\mathrm{Vd}\times \mathrm{MIC}}\right]\times \left[\frac{t_{1/2}}{0.693}\right]\times \left[\frac{100}{\mathrm{DI}}\right]$$

where: ln—natural logarithm, Vd—volume of distribution (L/kg), (kg), t_1/2_—serum half-life (hours), elimination rate constant (h-), DI = dosing interval (hours).

**Figure 2:**
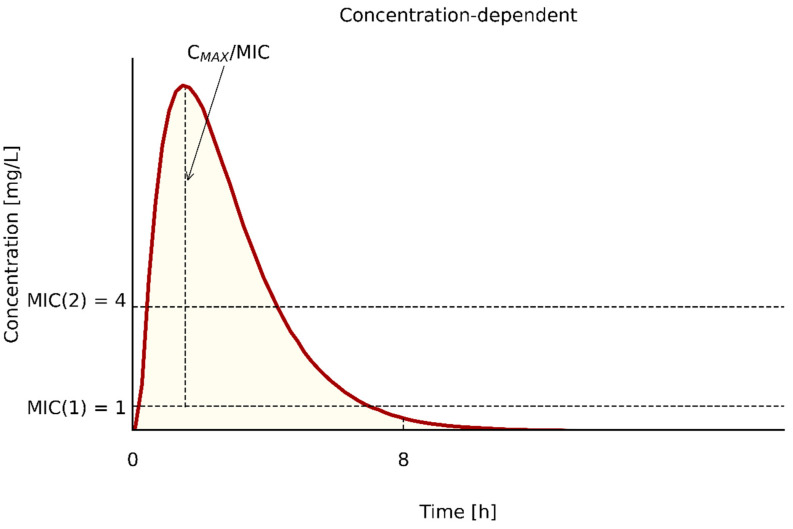
The relationship between C_max_ and MIC.

**Figure 3:**
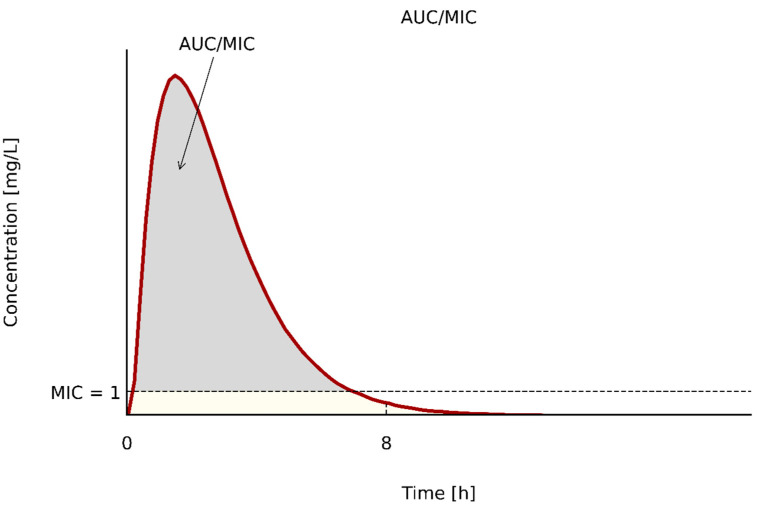
The relationship between AUC and MIC.

(2) Cmax/MIC is a parameter characteristic for antibiotics whose effectiveness depends on maximum concentration which is many times greater than the MIC (min. 8–10×) and not on the time it is kept above the MIC [58,64,73]. However, as with the previously discussed parameter, lower MIC values are more likely to meet the efficacy condition for these antibiotics while reducing the risk of toxic concentrations. The relationship between Cmax and MIC is shown in Figure 2.

(3) AUC/MIC (∫AUC/MIC): characterizes time- and concentration-dependent antibiotics [58,60,64,70,71,72,73,74,75,76]. As with the two previous parameters, the MIC value will influence drug effect. The relationship between AUC and MIC is shown graphically in Figure 3.

The formula for the calculation of the AUC/MIC index taking into account the MIC value [59] is as follows:

(2) $$\frac{\mathrm{AUC}}{\mathrm{MIC}}\;=\ln\left[\frac{\mathrm{Dose}}{\mathrm{Vd}\;\times \;\mathrm{MIC}}\right]\times \left[\frac{t_{1/2}}{0.693}\right]\times \left[\frac{24}{\mathrm{DI}}\right]$$

where: ln—natural logarithm, Vd—volume of distribution (L/kg), t_1/2_—serum half-life (hours), DI—dosing interval (hours).

The distribution of antibiotics according to PK/PD parameters is given in Table 4.

When using PK parameters in predicting the clinical efficacy of antibiotics, it is necessary to choose those that are used for specific groups of patients (ICU, nosocomial pneumonia, skin and soft tissue infections, intra-abdominal infections, children/adults, pregnant women, etc.) or to rely on those individually determined for a given patient. However, the latter option is much more difficult [77]. Automatic analyzers based on the immunoassay technique are available only for vancomycin and aminoglycosides to measure concentrations in the patient’s serum [77]. Therefore, usually these parameters are determined for populations. Unfortunately, the ones that are generally available have been established on the basis of studies of healthy people. Much more significant are PK factors determined for sick people because it is such patients who develop the most severe infections and whose treatments fail most often. The determination of the MIC value of most antibiotics is easier and more accessible, but it involves a certain problem. In order to determine the actual PK/PD index, the result of the MIC measurement should be known at the same time as the serum antibiotic concentration after administration, i.e., on the same day, which is virtually impossible. This requirement is dictated by the high dynamics of changes in drug concentrations over time after administration, while the MIC is a static value. Accelerating the determination of MIC would be very valuable for optimal therapy, similarly as it is currently possible to directly test drug susceptibility of positive blood cultures using the disc-diffusion method [78]. At one time, recognition was gained by direct methods of MIC determination in positive blood cultures using the strip-gradient method [79,80,81]. Ultimately, however, they have not obtained the approval of EUCAST or CLSI.

Currently, in anticipation of an individually determined MIC value, the MIC_90_ value, obtained on the basis of cumulative epidemiological analyses, may be applied. Due to problems related to PK determination in individual patient, statistical estimation of the possibility of achieving the PK/PD index based on the Monte Carlo method has gained recognition. The simulation uses population pharmacokinetic (PK) parameters specific to the patient groups concerned and the individual MIC values of antibiotics. The Monte Carlo method allows to approximate the relationship between the antibiotic MIC and the possibility of obtaining an optimal PK/PD index taking into account the specific dose of the medicine. It is also a useful tool that can be used to check if changing the dosage at the same MIC value will have a positive effect on the PK/PD index.

Such ready-made simulations are included among others in EUCAST recommendations in the Rationale documents [82]. However, these schemes need to be updated to take account of new knowledge on PK parameters obtained under different clinical conditions as well as of changing dosing rules.

## 5. Limitations Related to the Use of MIC Values

Drug sensitivity tests, including quantitative MIC tests, are based on the direct interaction of the pathogen isolated from the patient with a chemical (such as the antibiotic). The result obtained in such test does not take into account the factors affecting the antibiotic in the patient’s body. Factors such as volume of distribution, albumin levels or organ failures (which have already been mentioned in this paper) and also the activity of the immune system and its various components, other therapeutic procedures such as nutrition, blood transfusion, or additional drugs can significantly affect the final effect of treatment involving an antibiotic to which the micro-organism is susceptible [83,84,85,86]. Thus, the strain’s susceptibility alone does not guarantee clinical success. Obviously, with a high degree of susceptibility of the strain and especially with a MIC value below ECOFF, the chances for microbiological and clinical success increase. The higher the MIC, the greater the risk of failure, even though the strain may have been classified as sensitive to the antibiotic being prescribed. Such a phenomenon occurs, for example, with regard to vancomycin and its activity against *Staphylococcus aureus*. According to many studies, the effectiveness of *Staphylococcus* spp. eradication under the influence of this glycopeptide decreases significantly at MIC ≥ 1 mg/L [87,88,89,90]. The MIC value gains clinical significance only in relation to pharmacokinetic parameters. The combined analysis of these parameters is not simple and requires knowledge in the field of microbiology, pharmacology and clinical medicine, and hence requires the cooperation of a microbiologist, pharmacologist, physician and even a nurse, who ultimately carries out the doctor’s orders. The calculation of the optimal dose of the drug taking into account PK/PD indices is much more complicated than the formulas presented in this paper would indicate; nor was it the subject of this review. Certainly, the determination of a strain’s resistance to a drug based on the MIC value is an indicator of clinical failure. However, the ability of laboratories (even the best ones) to accurately assess the MIC value is being questioned. It turns out that a MIC assessment repeated twice, even by the same laboratory staff member, can produce MIC values differing up or down by 200% [85]. As Doern et al. explain, MIC = 2 mg/L may in reality mean 1 or 4 mg/L, which can result in both incorrect determination of sensitivity or resistance category and in predicting effectiveness based on PK/PD and determination of modified doses [85]. Among big drawbacks of MIC determinations is that the tests are performed for a specific bacterial inoculum. The result is therefore also representative of this standardized inoculum. If the number of bacteria is greater at the site of infection, despite the strain’s sensitivity determined in vitro, under in vivo conditions, the pathogen will prove resistant and the therapy will not be effective [83]. It may also happen that, with a low inoculum, the antibiotic may prove effective despite the fact that the strain has been determined to be resistant to it [84]. The effectiveness of therapy may also depend on the strain’s virulence, which is not reflected in the determined MIC value [85].

Another problem is the time required to obtain the result of drug sensitivity determination which is usually 3–5 days. Thus, the time at which the result is obtained is not the same as the time at which the test was ordered. As highlighted by Doern et al. [85], while waiting for the result, the patient is subjected to various procedures as a result of which the pathogens and their sensitivity may change. However, this certainly does not mean that drug sensitivity testing is useless because, as has already been mentioned in this paper, the knowledge of cumulative MIC values for specific pathogens is of significant value at the time therapeutic decisions are made. It can be used there and then both to calculate the PK/PD index and it may serve as a tool in formulating a rational antibiotic policy. It is important and emphasized in world literature and in numerous national and regional recommendations that MIC cumulative data should be determined only for bacteria derived from infections and not from colonization or contamination. Otherwise they will give false grounds for therapy optimization.

## 6. Conclusions

The MIC value is currently the best available parameter to reflect the effectiveness of an antibiotic against bacterial strains. Despite standardization of approved methods, it should be taken into consideration that the actual MIC value may differ ± double dilution from the one obtained in investigation. This difference usually does not affect clinical interpretation; although, with MIC value equal to breakpoint, it may be of high significance—firstly, to assess whether the strain is resistant or susceptible, and secondly, to use this value to optimal treatment selection involving PK/PD parameters.

The use of MIC value in treatment, based on individually defined PK parameters can significantly improve the effectiveness of antibiotic therapy. Although, the methodology for the determination of these parameters is very difficult and not even accessible to doctors, the PK/PD parameters can be estimated using the Monte Carlo method. Such a solution was proposed by EUCAST, to evaluate the achievement of PK/PD index depending on the MIC and antibiotic dose of known PK. In addition, recently, EUCAST, by introducing two susceptibility categories, has clearly made dose volume and road of administration depend on MIC values. The possibility to use the MIC values to increase the probability of therapeutic success can therefore be easier to implement. However, this does not change the fact that the attempt of determining this value in a microbiological laboratory is difficult due to methodology recommended by EUCAST and CLSI. According to these institutions, the most reliable is the broth microdilution method, a manual, demanding and time-consuming procedure. It should also be underlined that sometimes the information about susceptible strain, which is a result of even as precise value as the MIC, does not necessarily have to be true. It is known that bacteria have mechanisms that are impossible to detect in laboratory routine such as heterogeneous resistance, tolerance or persistence. Unfortunately, this feature of micro-organisms can contribute to the treatment failure, despite the optimal choice of antibiotic therapy.

## Figures and Tables

**Table 1:** Media, additional supplementation and control strains for MIC determination by the dilution methods according to EUCAST [3].

Bacterial Strains	Determination of MIC					Control Strains
	Broth Dilution			Agar Dilution		
	Mueller-Hinton Broth (MHB)	MHB + Defibrinated Horse Blood and β-NAD (MH-F)	Additional Supplementation	MHA	Additional Supplementation	
*Enterobacterales*	all antibiotics except fosfomycin and mecillinam	-	-	fosfomycin	MHA + 25 mg/L glucose-6-phosphate	*E. coli* ATCC 25922, inhibitors only: ATCC *E.coli* 35218 or *K. pneumoniae* 700603
*Enterobacterales*	all antibiotics except fosfomycin and mecillinam	-	-	mecillinam	-	*E. coli* ATCC 25922, inhibitors only: ATCC *E.coli* 35218 or *K. pneumoniae* 700603
*Pseudomonas* spp.	all antibiotics except fosfomycin	-	-	fosfomycin	MHA + 25 mg/L glucose-6-phosphate	*P. aeruginosa* ATCC 27853
*Stenotrophomonas maltophilia*	co-trimoxazole	-	-	-	-	*E. coli* ATCC 25922
*Acinetobacter* spp.	all antibiotics	-	-	-	-	*P. aeruginosa* ATCC 27853
*Staphylococcus* spp.	all antibiotics except fosfomycin	-	MHB + 2% NaCl for oxacillin, methicillin, nafcillin	fosfomycin	MHA + 25 mg/L glucose-6-phosphate	*S. aureus* ATCC 29213
*Staphylococcus* spp.	all antibiotics except fosfomycin	-	MHB + 50 mg/L Ca++ for daptomycin	-	-	*S. aureus* ATCC 29213
*Staphylococcus* spp.	all antibiotics except fosfomycin	-	MHB + 0.002% polysorbate 80 for dalbavancin, oritavancin, televancin	-	-	*S. aureus* ATCC 29213
*Enterococcus* spp.	all antibiotics	-	-	-	-	*E. faecalis* ATCC 29212
*Streptococcus A,B, C,G gr.*	-	all antibiotics	MH-F broth + 0.002% polysorbate 80 for dalbavancin, oritavancin, televanci	-	-	*S. pneumoniae* ATCC 49619
*Streptococcus pneumoniae*	-	all antibiotics	-	-	-	*S. pneumoniae* ATCC 49619
*Streptococcus gr. viridans*	-	all antibiotics	MH-F broth + 0.002% polysorbate 80 for dalbavancin, oritavancin, televanci	-	-	*S. pneumoniae* ATCC 49619
*Haemophilus* *influenzae*	-	all antibiotics	-	-	-	*H. influenzae* ATCC 49766
*Moraxella* *catarrhalis*	*-*	all antibiotics	-	-	-	*H. influenzae* ATCC 49766
*Listeria* *monocytog* *enes*	*-*	all antibiotics	-	-	-	*S. pneumoniae* ATCC 49619
*Pasteurella* *multocida*	*-*	all antibiotics	-	-	-	*H. influenzae* ATCC 49766
*Corynebacterium* spp.	*-*	all antibiotics	-	-	-	*S. pneumoniae* ATCC 49619
*Kingella kingae*	*-*	all antibiotics	-	-	-	*H. influenzae* ATCC 49766
*Aeromonas* *sanguinicola and urinae*	*-*	all antibiotics	-	-	-	*P. aeruginosa* ATCC 27853

“-“—not applicable.

**Table 2:** Solvents and diluents for different antibiotics [6].

Antibiotics		Solvent	Diluent
penicillins	penicillin, methicillin, nafcillin, oxacillin, azlocillin, mecillinam, mezlocillin, carbenicillin, piperacillin	water	
	amoxicillin, ticarcillin	phosphate buffer pH 6.0, 0.1 mol/L	
	ampicillin	phosphate buffer pH 8.0, 0.1 mol/L	phosphate buffer pH 8.0, 0.1 mol/L
beta-lactam inhibitors of beta-lactamases	sulbactam, tazobactam,	water	
	clavulanic acid,	phosphate buffer pH 6.0, 0.1 mol/L	
non beta-lactam inhibitors of beta-lactamases	avibactam, relebactam	water	
cephalosporins	cefaclor, cefamandole, cefonicid, cefoperazone, cefotaxime, cefoxitin, ceftozoxime, ceftoplozane, ceftriaxone	water	
	cefazolin, cefepime, cefuroxime	phosphate buffer pH 6.0, 0.1 mol/L	
	ceftazidime	sodium carbonate	Water
	ceftaroline	DMSO	Saline
	cephalexin, cephalotin, cephradine	phosphate buffer pH 8.0, 0.1 mol/L	water
carbapenems	faropenem, meropenem	water	
	ertapenem	phosphate buffer pH 6.0, 0.1 mol/L	
	imipenem, ertapenem	phosphate buffer pH 7.2, 0.01 mol/L	
	meropenem-varborbactam	DMSO	water
aminoglycosides	amikacin, gentamicin, kanamycin, netilmicin, streptomycin, plazomicin, tobramycin	water	
lincosamides	clindamycin	water	
macrolides	azitromycin	95% etanol	broth medium
	clarythromycin	methanol	phosphate buffer pH 6.5, 0.1 mol/L
	erythromycin	95% etanol	water
quinolones	cinafloxacin, finafloxacin, garenoxacin, gatifloxacin, gemifloxacin, moxifloxacin, sparfloxacin, ofloxacin *, levofloxacin *, norfloxacin *	water	
tetracyclines	tetracycline, minocycline, doxycycline, tigecycline, eravacycline	water	
polymyxins	colistin, polymyxin B	water	
glycopeptides	teicoplanin, vancomycin	water	
	telavancin	DMSO	
lipoglycopeptides	dalbavancin	DMSO	
cyclic lipopeptide	daptomycin	water	
oxazolidinones	linezolid	water	
	tedizolid	DMSO	
other antibiotics	fosfomycin, fusidic acid, mupirocin, quinupristin-dalfopristin,	water	
	fidaxomicin, metronidazole	DMSO	water
	chloramphenicol	95% ethanol	water
	rifampicin	methanol	water

* 1/2 volume of water, then 0.1 mol/L NaOH dropwise to dissolve, DMSO—Dimethyl sulfoxide.

**Table 3:** Antibiotics and pathogens for which susceptibility can only be derived from quantitative phenotype methods.

Antibiotic(s)	Group of Bacteria
fosfomycin	*Enterobacterales* except *E. coli*, *Staphylococcus* spp.
tigecycline	*Enterobacterales* except *E. coli*, *Citrobacter koserii*
colistin	all Gram-negative rods
all antibiotics	*Neisseria* spp., anaerobes
beta-lactams	penicillin nonsusceptible *Streptococcus pneumoniae*
glycopeptides/ lipoglycopeptides	*Staphylococcus* spp.
dalbavancin, oritavancin	*Streptococcus group: Viridans, A,B,C,G*

**Table 4:** Type of PK/PD ratio and target value for clinical efficacy.

PK/PD Type	Antibiotics	PK/PD Index	References
T > MIC	penicillins	≥50	[58,60,64,69]
	cephalosporins	≥50–70	[58,60,64,69]
	carbapenems	≥40	[58,60,64,69]
	for patients with immunosupresion	100	[60,65,66]
Cmax/MIC	aminoglycosides	>8	[58,64]
	fluoroquinolones	>8	[58,64]
	polymyxins	∫Cmax/MIC ≥ 6	[73]
	metronidazole	NK	
AUC/MIC	aminoglycosides	>70 [47]; ≥156 [41]	[58,64]
	ciprofloxacin	AUC/MIC > 125; ∫AUC/MIC > 88	[60]
	levofloxacin	AUC/MIC > 34; ∫AUC/MIC > 24	[60]
	vancomycin	AUC/MIC > 400; ∫AUC/MIC > 200	[60]
	daptomycin	AUC/MIC 388–537 [47]; AUC/MIC ≥ 666 [57]	[64,74]
	oksazolidinones	>80	[58,64,74]
	polymyxins	total AUC/MIC > 50; ∫AUC/MIC > 25	[64,73]
	fosfomycin	>8.6	[64]
	tygecycline	skin and skin structure infections AUC/MIC ≥ 17.9	[76]
		Intra-abdominal infections AUC/MIC ≥ 6.96	[76]
		hospital acquired pneumonia AUC/MIC ≥4.5	[76]
